# Incidence of Deep Vein Thrombosis in Patients With COVID-19: A Systematic Review

**DOI:** 10.7759/cureus.88697

**Published:** 2025-07-24

**Authors:** Daniela Secades, Sebastián Dufner Krieger, Roberto A Hidalgo Ramos, Isaac Hong, Marcelo Ortiz, Christopher Mac Courtney

**Affiliations:** 1 Medicine, University of Costa Rica, San Jose, CRI; 2 Medicine, University of Costa Rica, San José, CRI; 3 Vascular Surgery, University of Costa Rica, San Jose, CRI

**Keywords:** coagulation dysfunction, coronavirus disease, d-dimer, deep vein thrombosis, venous thromboembolism (vte)

## Abstract

COVID-19 is a contagious respiratory infection. This disease affected the healthcare system globally due to its complications related to vascular morbidity and mortality. There is a pathophysiological connection between COVID-19 and deep vein thrombosis (DVT). The blood hypercoagulability, venous stasis, and endothelial dysfunction in patients with COVID-19 have been associated with a higher risk of developing DVT. In this systematic review, 15 studies were selected to determine if COVID-19 can be taken as an independent risk factor for DVT. The search was done to determine studies analyzing the incidence of DVT in patients with COVID-19. The studies chosen were observational studies between 2020 and 2025.

## Introduction and background

COVID-19 affected more than 770 million people and caused approximately seven million deaths globally. COVID-19 is a highly contagious respiratory infectious disease caused by the severe acute respiratory syndrome coronavirus 2. Its clinical manifestation may vary from an asymptomatic or mild presentation to pneumonia or severe acute respiratory distress syndrome [1]. 

This virus has raised public concern in the health department due to its vascular complications. It has been associated with deep vein thrombosis (DVT), pulmonary embolism, and arterial thrombosis. Several studies have investigated the incidence of venous thromboembolism in patients with COVID-19, reporting rates as high as 14.7% [1].

DVT is an intravascular coagulation, or when a blood clot occurs in the deep system of veins of the body [1]. The most important risk factors for DVT are a history of a previous venous thromboembolism, the presence of a neoplasia, thrombophilias, pregnancy, smoking, travel that involves long distances, immobilization, and obesity [2].

Virchow’s Triad describes the components involved in the pathophysiology of DVT: hypercoagulability, venous stasis, and endothelial dysfunction. COVID-19 poses a risk for DVT because it generates all of these conditions. This study aimed to determine the association between COVID-19 and DVT [3]. 

Endothelial dysfunction is seen in COVID-19 when the virus infects endothelial cells through the angiotensin-converting enzyme 2, leading to a malfunction in the enzyme and causing the accumulation of angiotensin II. This generates an injury on the vascular endothelial cells because it leads to the expression of adhesion molecules (like tissue factor and plasminogen activator inhibitor type 1) and reduces the anticoagulant function of thrombomodulin. These changes facilitate the formation of vascular thrombus by creating a hypercoagulability environment. At the same time, they generate more inflammation that contributes to more plasma concentrations of fibrinogen and plasminogen activator inhibitor type 1 [3].

The inflammatory response and the platelets trigger the release of neutrophil extracellular traps (NETs) and an increase in neutrophil count. These NETs also disrupt the epithelial barrier. Cell-free DNA and myeloperoxidase (MPO)-DNA correlate with the number of neutrophils; so, in the COVID-19 inflammation that predisposes to DVT, they appear increased [4]. 

As well, interleukins and interferon type I are elevated in the proinflammatory state of COVID-19, increasing the risk for DVT. Interferon type I and virus RNA strands can cause the entry of the coronavirus into the cytoplasm of macrophages and activate an inflammasome. This leads to a secretion of interleukin-1-beta and interleukin-8, which activate macrophages and cause more procoagulant effects [4].

On the other hand, during the inflammation, endogenous anticoagulant components (such as tissue factor pathway inhibitor, antithrombin, and protein C) are reduced, leading to an even more hypercoagulable state [4].

The objective of this systematic review is to determine the incidence of DVT in this hypercoagulable state of COVID-19. 

## Review

Methods

**Figure 1:**
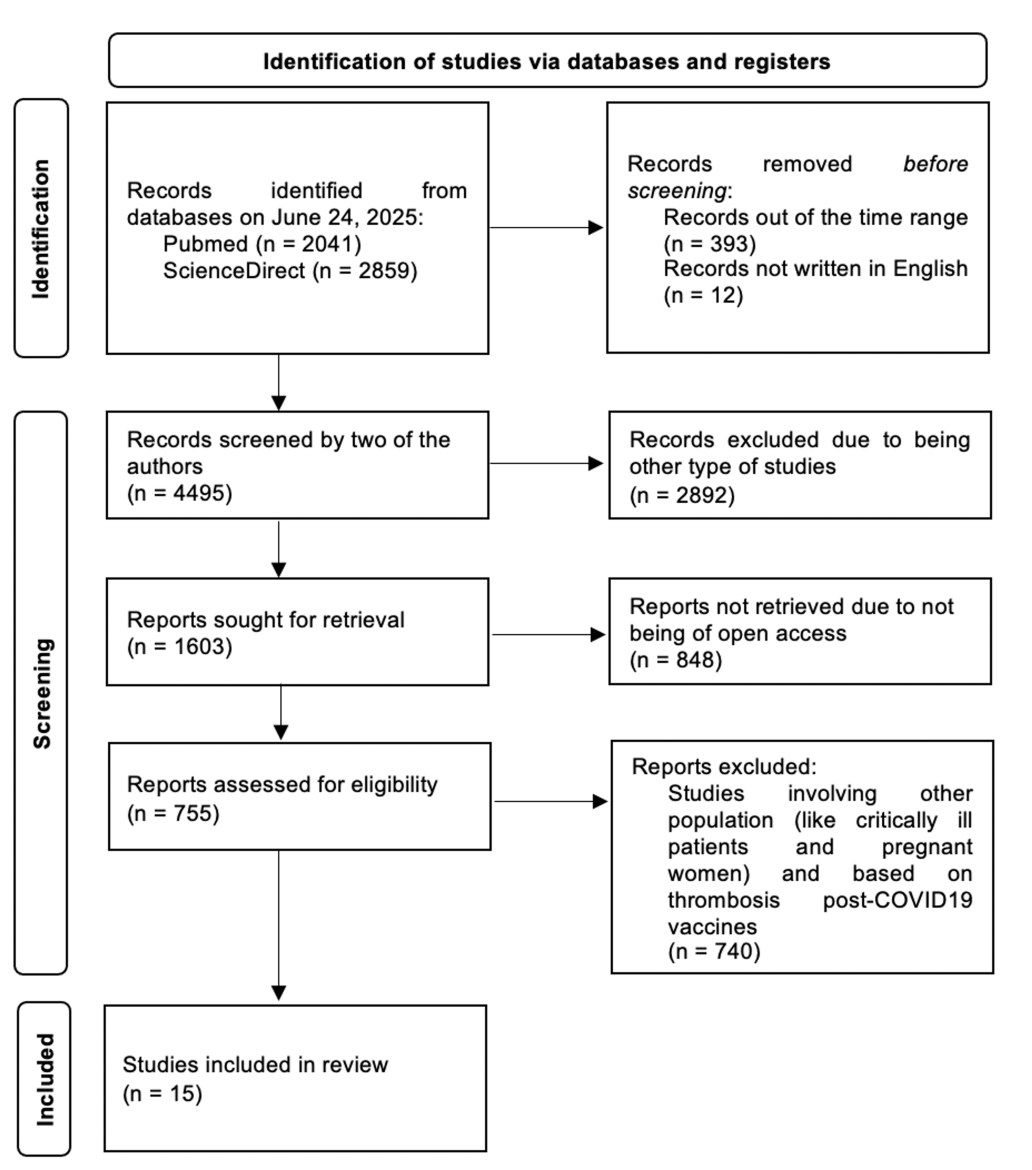
A PRISMA flowchart of the systematic review done to study the incidence of deep vein thrombosis in COVID-19 patients Preferred Reporting Items for Systematic Reviews and Meta-Analyses (PRISMA)

This review was developed according to the Preferred Reporting Items for Systematic Reviews and Meta-Analysis (PRISMA) guidelines of 2020 [5]. The databases used were PubMed and ScienceDirect. The research was based on the keywords “COVID-19” AND “Deep Vein Thrombosis,” and it was done on June 24, 2025. On the initial search, a total of 4,900 articles were obtained. Of these, all those studies that were not written in English, were duplicated, or were published before 2020, were excluded. Afterwards, a manual screening process was done, according to full-text availability, title, and inclusion and exclusion criteria, so the results were reduced to a total of 15 articles, as shown in Figure 1.

The search was done to determine studies analyzing the incidence of DVT in patients with COVID-19. The studies chosen were those between 2020 and 2025.

Inclusion Criteria

The criteria used to select the studies were articles written in English and conducted on humans, dates of publication between 2020 and 2025, full texts, and articles based on case-control, cohort, and observational studies.

Exclusion Criteria

The articles that were excluded were those published in another language, published before 2020, based on case reports, research articles, or systematic reviews; the non-full-text articles available; those involving other populations that were not adults; and those based on other types of thrombosis that were not DVT or on the effects of COVID-19 vaccines.

Risk of Bias

The risk of bias was determined by two of the authors using the Newcastle-Ottawa Quality Assessment Scale of Studies (NOS). The NOS establishes a star system that ranges from a total of 0 to nine points, based on three major components: selection of the population studied, comparability between the groups, and the ascertainment of the outcome of interest for cohort studies. According to this scale, a very good study is equal to nine points, a good study has a total of seven to eight points, a satisfactory one is represented by a total of five to six points, and an unsatisfactory study ranges from 0 to four points [6].

Results

A total of 4,900 articles were found: from ScienceDirect and from PubMed. Of those, 4,145 studies weren’t within the time frame chosen between 2020 and 2025, weren’t written in English, were systematic reviews, meta-analyses, or other types of studies other than the ones chosen, or weren’t open access, so they were excluded from the investigation. Afterwards, the studies were manually screened according to their titles and abstracts, so the search was reduced to a total of 15 articles included in this review.

Out of these 15 articles, 11 were retrospective studies, and four were prospective studies. Nine of them involved a population of the United States of America, two were from Spain, two were from China, and two were from Italy.

The quality of the information and the risk of bias were established with the NOS scale. According to this one, of the 15 studies analyzed, a total of one was seen as a very good study, 11 were good ones, and three were satisfactory. These findings are represented in Table 1 [6].

**Table 1:** Risk of bias of the 15 studies analyzed according to the Newcastle-Ottawa Quality Assessment Scale of Studies According to this scale, a very good study is equal to nine points, a good study has a total of seven to eight points, a satisfactory one is represented by a total of five to six points, and an unsatisfactory study ranges from 0 to four points [6].

	Lead author	Selection			Comparability		Outcome			Total
		Representativeness	Selection exposed cohort	Ascertainment	Result not present at the start of the study	Comparability for confounders	Assessment of outcome	Follow-up duration	Adequacy of follow-up	
1	Mei W et al., 2024 [1]	*	*	*	*	*	*	*	*	8
2	Koleilat I et al., 2021 [2]	*		*	*		*	*	*	6
3	Rali P et al., 2021 [7]	*	*	*	*	*	*	*	*	8
4	Jimenez-Guiu X et al., 2021 [8]	*	*	*	*	*	*	*	*	8
5	Erben Y et al., 2023 [9]	*	*	*	*		*	*	*	7
6	Choi J et al., 2020 [10]	*	*	*	*		*	*	*	7
7	Al-Samkari H et al., 2020 [11]	*	*	*	*		*	*	*	7
8	Motaganahalli R et al., 2021 [12]	*	*	*	*		*	*	*	7
9	Marini C et al., 2022 [13]	*	*	*	*	*	*	*	*	8
10	Thondapu V et al., 2021 [14]	*	*	*	*	*	*	*	*	8
11	Li J et al., 2021 [15]	*	*	*	*	**	*	*	*	9
12	Avruscio G et al., 2020 [16]	*	*	*	*		*	*	*	7
13	Franco-Moreno A et al., 2020 [17]	*		*	*		*	*	*	6
14	Pancani R et al., 2020 [18]	*		*	*		*	*	*	6
15	Brosnahan S et al., 2021 [19]	*	*	*	*	*	*	*	*	8

Discussion

DVT has a significant incidence in COVID-19 patients. The importance of studying this association lies in the fact that DVT has a significant impact in terms of the outcomes of the patients; it elevates the time that critically ill patients rely on mechanical ventilation, it increases the time that people spend in the hospitals, and it influences the morbidity and mortality of the patients [2].

In this systematic review, 15 articles were studied, and it was confirmed that there is a relationship between COVID-19 and DVT. The incidence of DVT in COVID-19 patients was between 3% and 47.5%, depending on the study. However, all of them stated that due to the hypercoagulability condition that coronavirus disease generates, it predisposes a greater risk for DVT.

The COVID-19 infection propagates an inflammasome-dependent pyroptosis of epithelial cells, where proinflammatory cytokines (interleukin-6, interferon-gamma, and interleukin-1-beta) are released, causing endotheliitis and liberation of the von Willebrand factor, facilitating the formation of thrombus. As well, the infection of the virus itself disrupts the endothelial cells [1]. This hypercoagulability, dysfunction in the endothelium, and venous stasis are the conditions that predispose COVID-19 as an independent risk factor for DVT.

Rali et al. determined that the incidence of venous thromboembolism in COVID-19 patients was 3.5% and the incidence of DVT was 9.5%. However, they recognize that their results might show a lower incidence rate than expected because the population under their study had received thromboprophylaxis. As well, they observed that admission D-dimer levels of more than 1500 ng/mL, intensive care unit admission, and the need for mechanical ventilation were associated with a higher risk of DVT in a COVID-19 context [7].

Another study that showed a similar prevalence was the one by Jimenez-Guiu et al., in which DVT was found in 10.5% of the COVID-19 patients. Their research is based on the hypercoagulable and inflammatory state generated when exposed to this respiratory disease, in which there is an increase in D-dimer, C-reactive protein, and von Willebrand factor and a decrease in antithrombin [8].

Along with an increase in D-dimer and the other risk factors associated with a higher prevalence of DVT, Erben et al. demonstrated that Black/African Americans have the highest risk of developing DVT when infected with COVID-19. This was shown with a 16.2% incidence of DVT in people belonging to this race affected by this respiratory disease. However, the number of patients taken into consideration in this article was mainly White patients (79.2%), and only 12.7% involved Black/African American patients, so there is a certain bias regarding these results [9].

Choi et al. revealed an incidence of 5.46% of DVT in COVID-19. As seen before, they observed that elevated D-dimer levels, Black race, need for supplemental oxygen, and higher platelet count were associated with a higher occurrence of DVT. They divided the levels of D-dimer according to low (when D-dimer is less than 1000 ng/mL), intermediate (1000-7500 ng/mL), and high (more than 7500 ng/mL) probability of suffering DVT in a COVID-19 context. As well, they also concluded that the Black race is the most vulnerable to DVT in COVID-19 because they have a higher prevalence of comorbidities (like obesity, diabetes, and hypertension), and they may have sickle cell traits [10].

This low rate of DVT in COVID-19 patients was also observed in the publication of Al-Samkari et al., where the results showed 4.8% of venous thromboembolism and 3% of DVT in COVID-19 patients. Nevertheless, they clarify that the results might have been underestimated because there was no uniform protocol to proceed with images in all patients with suspected venous thromboembolism and because thrombotic events may not have been diagnosed [11].

On the other hand, Motaganahalli et al. stated that the incidence of DVT in COVID-19 patients was as high as 37%. They observed that elevated D-dimers and alkaline phosphatase were strong predictors of DVT in COVID-19 patients. According to this, they developed an equation for the probability of DVT in female COVID-19 patients, which is as follows [12]:

$Pr(DVT=1)=\frac{e^{(-2.672+0.0002*ddimer-0.0013*CRP+0.0004*Ferritin+0.0269*AlkPO4-0.0042*Age-1.2947)}}{1+e^{(-2.672+0.0002*ddimer-0.0013*CRP+0.0004*Ferritin+0.0269*AlkPO4-0.0042*Age-1.2947)}}$

As well, they created the following equation for the probability of DVT in male COVID-19 patients* *[12].

$Pr(DVT=1)=\frac{e^{(-2.672+0.0002*ddimer-0.0013*CRP+0.0004*Ferritin+0.0269*AlkPO4-0.0042*Age)}}{1+e^{(-2.672+0.0002*ddimer-0.0013*CRP+0.0004*Ferritin+0.0269*AlkPO4-0.0042*Age)}}$

Marini et al. compared the incidence of DVT in COVID-19 patients versus the risk of DVT in those who didn’t suffer from the infection. They concluded that DVT was seen in 33.8% of the cases that tested positive for COVID-19 and in 12.4% of the non-COVID-19 ones. This conclusion reveals that there is a higher risk of DVT in COVID-19 [13].

The findings of the 15 studies reviewed are summarized in Table 2.

**Table 2:** Summary of the 15 studies included in this systematic review and their most important findings regarding deep vein thrombosis (DVT) in COVID-19 patients PE: pulmonary embolism

	Lead author	Country	Type of study and population	Findings	Conclusion
1	Mei W et al., 2024 [1]	China	Retrospective cohort study on adult patients admitted to the hospital in China between 2019 and 2023	Venous thromboembolism increased from 2019 to 2022 and reached its highest point during December 2022 and January 2023, when there was more liberty during the pandemic. The thromboembolism number was related to those COVID-19 patients who tested positive. The incidence of DVT in COVID-19 patients was 21% in hospitalized patients.	The number of cases of DVT increased when more people tested positive for COVID-19. There was a 21% incidence of DVT in COVID-19 patients.
2	Koleilat I et al., 2021 [2]	United States of America	Retrospective case-control study with patients admitted to the Montefiore Medical Center between March 1, 2020, and April 10, 2020 (n=135)	The incidence of DVT in COVID-19 patients was 13.3%. There was no difference regarding the need for mechanical ventilation, the ratio of arterial partial pressure of oxygen, the fraction of inspired oxygen, and the rate of acute kidney injury between the COVID-19 patients with DVT and the ones without it. However, there was a significant variation in the levels of D-dimer and fibrinogen; D-dimer in patients with DVT was found to be 18.88 µg/mL and fibrinogen of 501.0 mg/dL, versus those non-DVT that had values of 2.55 µg/mL of D-dimer and 654.5 mg/dL of fibrinogen. DVT occurs in COVID-19 patients, even when they have thromboprophylaxis.	Elevated D-dimer is important in studying DVT in COVID-19 patients. The hypercoagulability in COVID-19 with elevated D-dimer and less elevated fibrinogen leads to a significant risk of DVT, despite thromboprophylaxis.
3	Rali P et al., 2021 [7]	United States of America	Retrospective cohort study on patients admitted to the Temple University Hospital between April 1, 2020, to April 27, 2020 (n=147).	The venous thromboembolism incidence rate of 3.5% in patients admitted due to COVID-19. COVID-19 patients with D-dimer admission levels of more than 1500 ng/mL (P=0.013) or in need of invasive mechanical ventilation should be tested for venous thromboembolism. Fourteen out of 147 patients tested for venous thromboembolism were suffering from acute DVT, which represents 9.5%.	There is an association of DVT with COVID-19. Patients with elevated D-dimer or in need of invasive mechanical ventilation should be tested for venous thromboembolism.
4	Jimenez-Guiu X et al., 2021 [8]	Spain	Prospective cohort study on patients admitted to a Spain hospital during April 2020 (n= 67).	The incidence of lower DVT in COVID-19 patients with intermediate or complete anticoagulation doses was 10.5% and, in patients with prophylactic dosages, 16.2%.	DVT has been found in non-critically ill COVID-19 patients with a significant incidence of 10.5%, and even with a higher prevalence in critically ill patients.
5	Erben Y et al., 2023 [9]	United States of America	Retrospective study based on patients with COVID-19 at the Jacksonville campus of the Mayo Clinic from March 11, 2020, to May 27, 2021 (n=876)	The incidence of DVT/PE in COVID-19 patients was 8.7% (P=0.03). The prevalence of DVT in COVID-19 patients is higher among Black/African American patients, which was shown with a 16.2% incidence in this group.	DVT has an important incidence in COVID-19 patients. Black/African Americans have a higher risk of developing DVT in a COVID-19 infection.
6	Choi J et al., 2020 [10]	United States of America	Retrospective cohort study of COVID-19 patients admitted at the New York-Presbyterian/Weill Cornell Medical Center and New York-Presbyterian/Lower Manhattan Hospital between 3 March 2020 and 15 May 2020 (n= 1739).	Venous thromboembolism was seen in 7% of the COVID-19 patients. A total of 136 venous thromboembolism events occurred, where 79 of them were DVT of the lower extremities and 16 cases were DVT of the upper extremities (a total of 5.46% of DVT). Increased levels of D-dimer were related to a higher prevalence of venous thromboembolism in COVID-19 patients.	D-dimer is associated with the risk of DVT in COVID-19. The incidence of DVT in COVID-19 patients was low.
7	Al-Samkari H et al., 2020 [11]	United States of America	Retrospective study of COVID-19 patients admitted to five partner healthcare institutions on the 8^th^ of April 2020 (n=400)	Elevation of D-dimer, platelet count, C-reactive protein, and erythrocyte sedimentation rate was a predictor of DVT in COVID-19. The incidence of venous thromboembolism in COVID-19 patients was 4.8%. DVT was found in 12 out of the 400 patients, which represents a 3% incidence.	The incidence of DVT in COVID-19 patients was low. Elevated D-dimer is a strong predictor of DVT in COVID-19 patients.
8	Motaganahalli R et al., 2021 [12]	United States of America	Retrospective cohort study on COVID-19 patients admitted to the Indiana University Academic Health Center between March 15 and April 14, 2020 (n=71)	DVT was seen in 37% of COVID-19 patients, predominantly in the male ones (67%, P=0.032). Patients with elevated D-dimer (mean of 5447 + 7032 ng/mL, P=0.0101) and alkaline phosphatase (110 UI, P=0.0095) correlated with a higher risk of DVT when suffering COVID-19.	There is a significant incidence of DVT in COVID-19 patients. Males are the ones with the highest risk for DVT. Elevated D-dimer and alkaline phosphatase are associated with a higher risk of DVT.
9	Marini C et al., 2022 [13]	United States of America	Retrospective cohort study at a level one trauma center comparing non-COVID-19 patients and those with the disease during March 20, 2019, and June 30, 2019 (n= 786, where 573 were non-COVID-19 patients and 213 were COVID-19 patients).	The incidence of DVT in COVID-19 patients was 33.8% whereas in non-COVID-19 patients that only 12.4% suffered from DVT. The elevation of D-dimer was associated with the incidence of DVT.	The incidence of DVT was higher in patients with COVID-19 than in those without it.
10	Thondapu V et al., 2021 [14]	United States of America	Retrospective cohort study on COVID-19 patients from March 13 to May 18, 2020 (n=138)	The incidence of venous thromboembolism in COVID-19 patients was 31.9% and specifically, of DVT, it was 20.2%. Patients with male sex, elevated C-reactive protein, and elevated platelet count at admission were the ones with the highest risk of venous thromboembolism in a COVID-19 context. Mortality in venous thromboembolism in COVID-19 patients increased in the presence of active malignancy, disseminated intravascular coagulation, and increased D-dimer.	COVID-19 is associated with DVT.
11	Li J et al., 2021 [15]	China	Retrospective cohort study of COVID-19 patients in 16 centers in China between January 1 to March 31, 2020 (n=2779)	Venous thromboembolism was seen in 5.94% of the severe hospitalized COVID-19 patients and in 2.79% of the non-severe ones. D-dimer had the highest association with DVT in COVID-19 patients. However, DVT and COVID-19 patients also had higher levels of white blood cell count, neutrophils, and C-reactive protein and lower levels of fibrinogen. In 104 of their venous thromboembolism cases, 88 were of DVT.	DVT is prevalent in COVID-19 patients. Elevated D-dimer is a strong predictor of DVT in COVID-19 patients.
12	Avruscio G et al., 2020 [16]	Italy	Prospective cohort study of COVID-19 patients admitted to the Padua University Hospital from March 4 to April 30, 2020 (n= 85)	Venous thromboembolism was seen in 27.3% of COVID-19 patients in the medical ward and in 75.6% in the intensive care unit (p<0.0001). DVT happened in 42.4% of the patients. D-dimer has a strong association with venous thromboembolism in COVID-19 patients.	DVT has a significant incidence in COVID-19. D-dimer is strongly related to DVT in COVID-19.
13	Franco-Moreno A et al., 2020 [17]	Spain	Prospective study with COVID-19 patients with pulmonary embolism at the Infanta Leonor University Hospital from March 30, 2020, to May 6, 2020 (n=26)	The incidence of DVT in COVID-19 patients with pulmonary embolism was 7.7%.	DVT had a 7.7% incidence in COVID-19 patients who had suffered from pulmonary embolism.
14	Pancani R et al., 2020 [18]	Italy	Prospective study of admitted COVID-19 patients in three pulmonary units in Italy between March 27 and May 6, 2020 (n=68)	The incidence of DVT was observed in 3% of the COVID-19 patients	DVT has a low incidence in COVID-19 patients.
15	Brosnahan S et al., 2021 [19]	United States of America	Retrospective study of patients in the New York University Langone Health Manhattan Campus with thrombosis and with or without COVID-19 from March 1, 2020, to April 30, 2020 (n=129)	D-dimer, white blood cell count, C-reactive protein, ferritin, bilirubin, and transaminases were higher in patients with DVT and COVID-19. Of 40 patients with COVID-19, 19 of them suffered from above-the-knee DVT, which represents 47.5%.	The incidence of DVT increased after the COVID-19 pandemic.

## Conclusions

In conclusion, DVT has been shown to have an incidence from 3% to 4.8% (as the lowest documented) and up to 47.5% (as the highest percentage seen in the studies) in patients with COVID-19. Most of the 15 studies reviewed in this systematic review suggested that there is a strong association between COVID-19 and DVT. As well, they determined that patients with elevated D-dimer were the highest predictor correlated to DVT in the context of COVID-19 due to the hypercoagulability and inflammatory state that this represents. The mortality surrounding DVT in COVID-19 patients is the main reason why studies should raise awareness on the importance of thromboprophylaxis and in determining the further relationship between DVT and COVID-19.
